# The intentions and factors influencing university students to perform CPR for strangers based on the theory of planned behavior study

**DOI:** 10.1016/j.heliyon.2024.e38135

**Published:** 2024-09-19

**Authors:** Lihua Xia, Kebiao Zhang, Feiyue Huang, Ping Jian, Runli Yang

**Affiliations:** a Department of emergency medicine, The First Affiliated Hospital of Chongqing Medical University, Chongqing, China; b Department of urology, The First Affiliated Hospital of Chongqing Medical University, Chongqing, China; c Department of nursing, Chongqing Nursing Vocational College, Chongqing, China

**Keywords:** University students, The theory of planned behavior, Cardiopulmonary resuscitation

## Abstract

**Objective:**

The Theory of Planned Behavior (TPB) was used to compile a questionnaire to determine the relationship between knowledge, attitude, subjective norm, perceived behavioral control and the intention of university students to perform cardiopulmonary resuscitation (CPR) for strangers, and the factors influencing them.

**Methods:**

We recruited 575 university students who completed an online questionnaire within 30 min to assess knowledge, attitude, subjective norm, and perceived behavioral control related to bystander CPR. Factor analysis was used to evaluate the reliability of the extended questionnaire. Multivariate analysis, correlation analysis, and path analysis were used to determine the differences of intra-group and inter-group.

**Results:**

The Cronbach's α of knowledge, attitude, subjective norm, perceived behavioral control and intention were 0.770, 0.797, 0.909, 0.619 and 0.899 respectively, indicating that the extended scale reliability of the TPB was adequate. χ2/df = 3.463, GFI = 0.977, CFI = 0.968, IFI = 0.969, RMSEA = 0.066, indicating that the extended scale of the TPB had a good fit. Path analysis showed that the influencing factors of intention were “Families of medical workers”, “Experience in administering CPR first aid training”, “Gender”, “Ability to master CPR skills”, “Knowledge”, “Subjective norm” and “Attitude”, with effect values of 0.019, 0.063, 0.069, 0.122, 0.187, 0.361 and 0.386, respectively. All the factors together accounted for 57.00 % of the variation in intention.

**Conclusions:**

Knowledge, attitude and subjective norm regarding to bystander CPR among university students are the determinants of intention, while perceived behavioral control does not play a major role. This study has implications for future CPR training. In order to improve the CPR implementation rate of university students, we should strengthen the relevant knowledge about CPR, maintain positive attitude and refine the related criteria of CPR implementation.

## 1 Introduction

Cardiac arrest, which is characterized by the abrupt cessation of heart function, is a serious medical emergency that is associated with high mortality globally [1,2]. In China, the incidence of sudden cardiac death (SCD) is alarmingly high, estimated at 40.7/100,000 people per year. Shockingly, about 70 % of these cases occur outside the hospital [[3], [4], [5]]. Survival rates for those who experience out-of-hospital cardiac arrest (OHCA) are generally low worldwide, ranging from 2 to 11 %; however, in China, this rate is even lower at only 1 % [6,7]. The etiology of SCD varies with age. In young and middle-aged populations, the main causes include primary heart diseases, cardiomyopathy, myocarditis, and coronary artery disease, but chronic structural diseases including valvular heart disease and heart failure for the elderly population [8]. Cardiopulmonary resuscitation (CPR), as a first aid technique, is used to restore heart rate and/or spontaneous breathing in a patient with cardiac and/or respiratory arrest by chest compressions and/or artificial respiration [9,10]. The timely recognition of cardiac arrest, the implementation of bystander CPR, and the use of automated external defibrillators (AEDs) are all critical factors impacting the survival and quality of life for individuals who experience sudden cardiac arrest [11,12]. Particularly in China, it is essential to raise general awareness of the importance of early intervention measures and to equip more people with the skills and tools needed to provide effective assistance during cardiac emergencies.

Studies have shown that the survival rate of cardiac arrest patients can reach up to 49%–75 % if they receive correct and effective CPR within the first 3–5 min [13]. This emphasizes the crucial need for bystanders to be trained and equipped with the knowledge to perform CPR in the event of an emergency. However, it is worrying to note that most spectators do not perform CPR to patients with OHCA for the first time [14]. This is particularly concerning as the first responders to cardiac arrest are often relatives, friends, colleagues, or even strangers around the patient. Unfortunately, the current penetration rate of adult CPR in China is less than 1 %, which is far below the global average, leaving many people unable to perform proper CPR on cardiac arrest victims [7,15]. In fact, data show that the rate of out-of-hospital CPR is only 4.5 % in mainland China, compared to 46.1 % in the United States, 29 % in Canada, 46–73 % in Sweden, 32.2 % in Japan, and 21.2 % in Australia [7]. It is clear that there is an urgent need for more widespread CPR education and training in China. Additionally, it is important to note that whether an eyewitness gives timely CPR to a sudden death victim depends not only on their level of knowledge about CPR, but more importantly on their beliefs and attitude to perform bystander CPR [16,17]. Therefore, it is important not only to provide CPR education, but also to address any potential barriers or misconceptions that may prevent individuals from performing bystander CPR.

As an important reserve of the social population and a high-quality group of nationals who are about to enter all walks of life, university students have a high degree of plasticity and enthusiasm for participating in society. Therefore, it is imperative to promote university students as performers for out-of-hospital CPR in order to promote the popularization of CPR and further improve CPR awareness and skills among university students. Huang et al. [18] found that only 14.6 % of university students had received CPR training, and almost all universities surveyed said they would perform bystander CPR on family members, while only 59.7 % said they would perform bystander CPR on strangers. Approximately 90 % of students were reluctant to perform bystander CPR on strangers due to lacking of information and fearing of failure. Lu et al. [19] further supported these findings by showing that students' intention to perform bystander CPR depended mainly on their own previous experience.

Panchal and colleagues proposed that the Theory of Planned Behavior (TPB) could be a useful tool for examining bystander intention to perform CPR [20]. TPB is a widely studied theory in the field of social psychology, which emphasizes the link between attitude and behaviors. According to this theory, behavioral intention directly determines individual behavior when the actual control conditions are sufficient. Behavioral intention itself is the result of the comprehensive effect of the three elements of attitude, subjective norm, and perceived behavioral control [21]. Researchers have successfully applied TPB to study health-related behaviors such as diet, exercise, and healthcare management [[22], [23], [24]].

According to the TPB, university students' intention to perform bystander CPR on strangers in cardiac arrest is the result of three factors: attitude, subjective norm, and perceived behavioral control. Innovatively, we applied the extended TPB to analyze university students' willingness to perform bystander CPR on strangers and its influencing factors in China, and then proposed countermeasures to improve university students' intention to perform bystander CPR.

## 2 Methods

1 Samples

From May to August 2021, we conducted an online questionnaire among full-time university students (including junior college students and undergraduates) aged 18 and above in China. Double entry was implemented to ensure the quality of the questionnaire, then, the logic of the original data was checked, the logical problems were corrected, and the sample data with outliers and more missing variables were eliminated. Finally, 575 valid samples were obtained, including 106 males and 469 females.

Informed consent was obtained from all participants and the questionnaire was completed within 30 min. The study received ethical approval from the First Affiliated Hospital of Chongqing Medical University.

2 Survey instrument

**Fig. 1:**
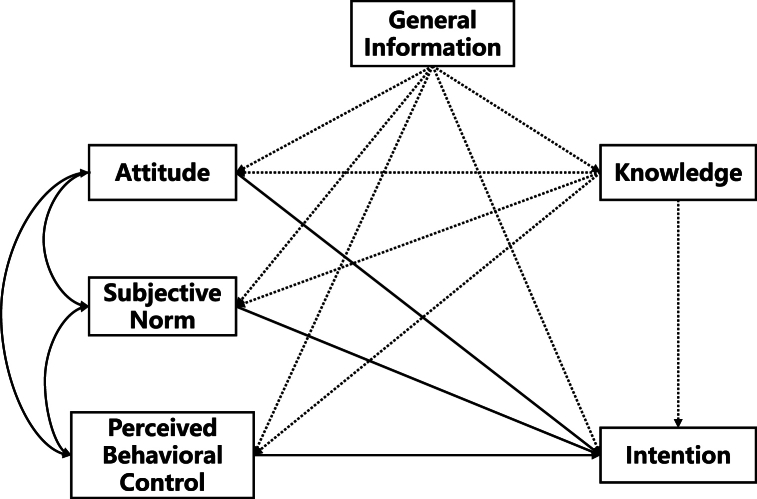
**Extended model diagram of the TBP.** The solid lines are the original paths and the dashed lines are the extended paths.

To investigate students' intention to provide bystander CPR on strangers and the factors influencing it, we innovatively included general information and knowledge according to the TPB questionnaire construction guide (http://openaccess.city.ac.uk/id/eprint/1735) (Fig. 1). The questionnaire was designed based on the scientific and operational principles of the literature induction method. All question options of the questionnaire were finalized by experts, then a small pre-survey was conducted to correct the possible deviations and to test the reliability of the questionnaire. Finally, a standardized formal questionnaire was created. The questionnaire covered the following three aspects:

### 2.1 General information questionnaire

We designed questions on general information such as age, gender, major, grade, educational background, as well as “whether you have relatives at high risk of SCD, such as coronary heart disease’’, “whether you have taken CPR training”, “whether you have seen strangers faint”, “whether you have observed CPR in the field”, “whether you are a family member of a medical professional”, and “how well you think you can do CPR”, etc.

### 2.2 CPR related knowledge questionnaire

We invited 5 CPR related experts to revise this part, all of whom had postgraduate degrees and had been involved in CPR teaching for more than 10 years. We modified this part after discussion based on the experts' opinions, and this part was revised twice in total. The purpose of this section was to understand the respondents' understanding about CPR. There were 11 questions, all true or false. For example, “Adult CPR procedures are: chest compressions, open airway and artificial respiration”, “CPR is appropriate for all cardiac arrest patients without contraindications”, etc.

### 2.3 Intention scale of bystander CPR for strangers in university students based on the TPB

Following the TPB questionnaire construction guide, 42 questions were designed to assess students' attitude, subjective norm and perceived behavioral control in relation to bystander CPR. A 7-point Likert scale was used to represent beliefs about performing CPR according to the TPB. The 7-point Likert scale was extremely anchored with poles including “strongly agree/strongly disagree”, “extremely likely/extremely unlikely”, “strongly reject/strongly accept”, and “pleasant/unpleasant”, etc. Examples of items were “If I perform bystander CPR on a stranger, I think I am doing something positive for them”, “I am afraid of doing it incorrectly and causing other complications such as broken ribs”, “It is entirely up to me whether I want to perform bystander CPR on a stranger”, etc.

3 Verification of the questionnaire

As shown in Table 1, the factor analysis of the TPB showed that the cumulative variance contribution of the four factors was 61.88 %, KMO = 0.931, and the chi-square value of the Bartlett spherical test was 20898.7499, P < 0.001, indicating that the validity of the scale was fair. The Cronbach's alpha of attitude, subjective norm, perceived behavioral control and intention were 0.797, 0.909, 0.619 and 0.899, respectively, indicating that the reliability of the scale was adequate.

4 Statistical description

**Table 1:** Factor analysis of the extended questionnaire based on the TBP.

Dimension	Cronbach's α	M ± SD	Min, Max
Knowledge	0.770	7.34 ± 1.57	0, 9
Attitude	0.797	65.06 ± 10.28	14, 98
Subjective norm	0.909	56.07 ± 10.83	17, 77
Perceived behavioral control	0.619	59.46 ± 8.1	31, 81
Intention	0.899	16.08 ± 4.14	3, 21

Note. Cronbach's alpha was used to determine the reliability of the extended scale. Cronbach's α > 0.60 means the reliability of the scale was adequate.

SAS version 9.4 was used for data collection and analysis. Measurement data with normal distribution were described by mean ± standard deviation (M ± SD), and comparison between groups was performed by two-sample *t*-test and ANOVA. Pearson correlation analysis was used to analyze the correlation between variables. Amos version 24.0 was used for path analysis, maximum likelihood estimation was used to estimate parameters, and the model was adjusted according to the correction index. When χ2/df < 5.0, root mean square error of approximation (RMSEA) < 0.08, goodness of fit index (GFI) ≥0.90, incremental fit index (IFI) ≥0.90 and confirmatory fit index (CFI) ≥0.90, the model fitted well. The confidence interval (CI) was calculated using the bias-corrected percentile bootstrap method with 2000 replicate samples. The inspection level for this study was set as α = 0.05.

## 3 Results

1 The mastery levels of CPR skills determine the intention of bystander PCR

A total of 575 students (including 107 junior college students and 468 undergraduates) participated in this survey. These students are from 13 departments, such as civil engineering school, resources and environment school, sporting academy, geography department, chemistry college, architecture and planning college, medical college and so on. 493 students study in universities located in city, and another 82 students study in rural. The relationship between general information, knowledge and different dimensions of the TPB is shown in Table 2. 66.43 % (382/575) of the students had received CPR first aid training, and there were statistical differences in knowledge and all dimensions. Similarly, the mastery levels of CPR skills determined the intention to perform bystander PCR. 75.83 % (486/575) of the students believed that they were proficient in CPR and could perform bystander CPR on a stranger. Moreover, there was statistical significance between the knowledge and the intention in the different gender. In addition, there was no statistical significance in the different dimensions of the surveyed students in different ages and educational background.

2 The acquisition levels of cardiopulmonary resuscitation skills are the main factor affecting the willingness

**Table 2:** The relationship between general information, knowledge and the dimensions of the TPB.

	Sample n (%)	Knowledge	Attitude	Subjective norm	Perceived behavior control	Behavioral intention
**Age**						
≤18	73 (12.70)	7.51 ± 1.64	64.15 ± 10.75	53.51 ± 12.25	58.96 ± 8.64	15.47 ± 4.24
19∼20	260 (45.22)	7.42 ± 1.35	64.8 ± 10.45	56.42 ± 9.69	59.67 ± 7.11	16.18 ± 3.92
≥22	242 (42.09)	7.21 ± 1.75	65.62 ± 9.95	56.48 ± 11.45	59.38 ± 8.92	16.15 ± 4.34
F value		1.513	0.727	2.361	0.233	0.914
P value		0.221	0.484	0.095	0.792	0.402
**Gender**						
Male	106 (18.43)	6.84 ± 2.26	63.65 ± 12.35	54.51 ± 12.71	58 ± 9.13	15.07 ± 4.71
Female	469 (81.57)	7.46 ± 1.34	65.38 ± 9.73	56.43 ± 10.34	59.79 ± 7.82	16.3 ± 3.97
t value		−2.717	−1.351	−1.448	−1.866	−2.513
P value		0.008	0.179	0.15	0.064	0.013
**Education**						
Junior college	107 (18.61)	7.47 ± 1.81	66.94 ± 11.95	56.05 ± 11.98	59.43 ± 8.22	16.19 ± 4.15
University	468 (81.39)	7.32 ± 1.51	64.63 ± 9.82	56.08 ± 10.56	59.46 ± 8.08	16.05 ± 4.14
t value		0.801	1.862	−0.028	−0.039	0.306
P value		0.425	0.065	0.978	0.969	0.760
**Relatives with high risk of SCD such as coronary heart disease**						
Yes	63 (10.96)	7.89 ± 0.79	67.94 ± 11.11	59.19 ± 12.09	61.68 ± 7.98	16.89 ± 4.02
No	512 (89.04)	7.28 ± 1.63	64.71 ± 10.13	55.69 ± 10.61	59.18 ± 8.08	15.98 ± 4.15
t value		4.999	2.361	2.432	2.320	1.653
P value		<0.001	0.019	0.015	0.021	0.099
**Experience in administering CPR first aid training**						
Yes	382 (66.43)	7.59 ± 1.14	65.82 ± 9.73	57.47 ± 9.88	60.63 ± 7.53	16.46 ± 3.76
No	193 (33.57)	6.86 ± 2.11	63.57 ± 11.16	53.31 ± 12.05	57.13 ± 8.69	15.31 ± 4.73
t value		4.485	2.378	4.150	4.771	2.950
P value		<0.001	0.018	<0.001	<0.001	0.003
**Experience in witnessing someone faint**						
Yes	158 (27.48)	7.53 ± 1.14	66.49 ± 10.47	57 ± 10.85	59.7 ± 7.77	16.51 ± 4.24
No	417 (72.52)	7.28 ± 1.7	64.52 ± 10.16	55.72 ± 10.81	59.36 ± 8.23	15.91 ± 4.09
t		2.028	2.052	1.264	0.446	1.534
P		0.043	0.041	0.207	0.655	0.126
**Experience in watching the scene of CPR**						
Yes	106 (18.43)	7.75 ± 1.32	66.86 ± 11.18	57.72 ± 11.78	60.95 ± 8.04	16.74 ± 3.88
No	469 (81.57)	7.25 ± 1.61	64.66 ± 10.03	55.7 ± 10.58	59.12 ± 8.08	15.93 ± 4.18
t value		3.323	1.997	1.734	2.111	1.819
P value		0.001	0.046	0.083	0.035	0.069
**Families of medical workers**						
Yes	95 (16.52)	7.77 ± 1.39	65.04 ± 12.24	57.45 ± 11.57	61.05 ± 8.8	16.25 ± 4.36
No	480 (83.48)	7.26 ± 1.59	65.07 ± 9.86	55.8 ± 10.67	59.14 ± 7.92	16.04 ± 4.1
t value		2.901	−0.018	1.360	2.108	0.454
P value		0.004	0.985	0.174	0.035	0.650
**Ability to master CPR skills**						
Expert	436 (75.83)	7.52 ± 1.3	65.86 ± 9.83	57.05 ± 9.83	60.33 ± 7.53	16.46 ± 3.63
Inexpert	139 (24.17)	6.81 ± 2.13	62.55 ± 11.25	53.01 ± 13.07	56.72 ± 9.18	14.88 ± 5.28
t value		3.718	3.118	3.349	4.208	3.292
P value		<0.001	0.002	0.001	<0.001	0.001

Note. SCD: sudden cardiac death; CPR: cardiopulmonary resuscitation.

We further performed multi-factor linear regression model by using factors with statistic difference in Table 2, and variables were screened using a stepwise method (Table 3, Table 4). We found that the level of CPR skills acquisition was the main factor influencing intention. In addition, the results of the correlation analysis showed a positive correlation between the two pairs of knowledge, attitude, subjective norm, perceived behavioral control and intention (see Table 5).

3 Knowledge, attitude and subjective norm are the direct influencing factors of intention

**Table 3:** Variable assignment.

Independent variable	assignment
Gender	male = 0, female = 1
Relatives with high risk of SCD such as coronary heart disease	no = 0, yes = 1
Experience in administering CPR first aid training	no = 0, yes = 1
Experience in witnessing someone faint	no = 0, yes = 1
Experience in watching the scene of CPR	no = 0, yes = 1
Families of medical workers	no = 0, yes = 1
Ability to master CPR skills	no = 0, yes = 1

Note. SCD: sudden cardiac death; CPR: cardiopulmonary resuscitation.

**Table 4:** Multifactor regression model analysis between general information, knowledge and the dimensions of TPB.

Dependent variable	Independent variable	β	Standard error	Standardized regression coefficient	T value	P value
**Knowledge**	Constant	6.255	0.175	–	35.639	<0.001
	Gender: female	0.445	0.167	0.11	2.673	0.008
	Relatives with high risk of SCD such as coronary heart disease	0.419	0.205	0.083	2.047	0.041
	Experience in administering CPR first aid training	0.471	0.147	0.142	3.204	0.001
	Families of medical workers	0.427	0.172	0.101	2.486	0.013
	Ability to master CPR skills	0.392	0.162	0.107	2.415	0.016
**Attitude**	Constant	61.639	0.92	–	67.015	<0.001
	Relatives with high risk of SCD such as coronary heart disease	2.686	1.363	0.082	1.971	0.049
	Ability to master CPR skills	3.403	0.99	0.142	3.435	0.001
**Subjective norm**	Constant	51.795	0.962	–	53.869	<0.001
	Relatives with high risk of SCD such as coronary heart disease	2.862	1.422	0.083	2.013	0.045
	Experience in administering CPR first aid training	3.002	1.029	0.131	2.918	0.004
	Ability to master CPR skills	2.597	1.129	0.103	2.3	0.022
**Perceived behavior control**	Constant	55.877	0.713	–	78.399	<0.001
	Experience in administering CPR first aid training	2.602	0.762	0.152	3.414	0.001
	Ability to master CPR skills	2.442	0.841	0.129	2.904	0.004
**Intention**	Constant	14.27	0.458	–	31.163	<0.001
	Gender:female	0.908	0.449	0.085	2.023	0.044
	Ability to master CPR skills	1.406	0.406	0.145	3.458	0.001

Note. SCD: sudden cardiac death; CPR: cardiopulmonary resuscitation; β: regression coefficient.

**Table 5:** Correlation analysis.

Dimension	M ± SD	Knowledge	Attitude	Subjective norm	Perceived behavior control	Intention
**Knowledge**	7.34 ± 1.57	1				
**Attitude**	65.06 ± 10.28	0.140∗∗∗	1			
**Subjective norm**	56.07 ± 10.83	0.249∗∗∗	0.704∗∗∗	1		
**Perceived behavior control**	59.46 ± 8.1	0.216∗∗∗	0.580∗∗∗	0.701∗∗∗	1	
**Intention**	16.08 ± 4.14	0.225∗∗∗	0.691∗∗∗	0.698∗∗∗	0.562∗∗∗	1

**Fig. 2:**
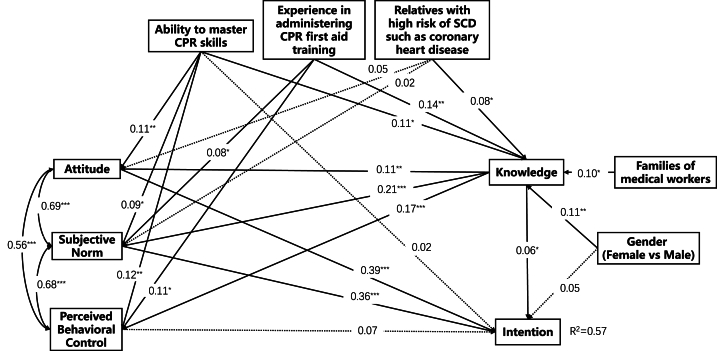
**Detailed and extended model diagram of the TPB.** The solid lines are P < 0.05 for path coefficients, where ∗P < 0.05, ∗∗P < 0.01, ∗∗∗P < 0.001; the dashed lines are P > 0.05 for path coefficients.

Next, we constructed the path analysis of the extended TPB model according to the results of the correlation analysis and the multiple factors analysis. As shown in Table 6, the path analysis model was well fitted, evidenced by the effect values of χ2/df 3.463, GFI 0.977, CFI 0.968, IFI 0.969 and RMSEA 0.066. Knowledge, attitude and subjective norm were the direct influencing factors of the intention, and knowledge could indirectly influence intention by influencing attitude and subjective norm, with the indirect effect size of 69.52 %. In addition, gender and the mastery of CPR-related skills have no direct effects on intention, while knowledge, attitude and subjective norm might indirectly affect intention, with the indirect effect sizes of 30.43 % and 84.43 % respectively. There was an indirect effect between family members of health care workers and experience with CPR training. The factors influencing intention include “having a family member who is a medical worker”, “experience in CPR training”, “gender”, “knowledge of CPR-related skills”, “knowledge”, “subjective norm” and “attitude”, with effect values of 0.019, 0.063, 0.069, 0.122, 0.187, 0.361 and 0.386 (Fig. 2).

**Table 6:** Total, direct, and indirect effect of factors influencing intention.

Index	Total effect (95%CI)	P value	Direct effect (95%CI)	P value	Indirect effect (95%CI)	P value	Indirect effect size
**Families of medical workers**	0.019 (0.006,0.041)	0.002	–	–	0.019 (0.006,0.041)	0.002	100.00 %
**Relatives with high risk of SCD such as coronary heart disease**	0.042 (-0.011,0.096)	0.115	–	–	0.042 (-0.011,0.096)	0.115	100.00 %
**Experience in administering CPR first aid training**	0.063 (0.033,0.1)	<0.001	–	–	0.063 (0.033,0.1)	<0.001	100.00 %
**Perceived behavior control**	0.066 (-0.024,0.152)	0.161	0.066 (-0.024,0.152)	0.161	–	–	–
**Gender: female**	0.069 (0.005,0.133)	0.036	0.048 (-0.017,0.106)	0.130	0.021 (0.003,0.052)	0.017	30.43 %
**Ability to master CPR skills**	0.122 (0.028,0.213)	0.015	0.019 (-0.048,0.085)	0.570	0.103 (0.039,0.166)	0.003	84.43 %
**Knowledge**	0.187 (0.081,0.289)	0.001	0.056 (0,0.113)	0.049	0.13 (0.047,0.212)	0.001	69.52 %
**Subjective norm**	0.361 (0.244,0.489)	0.001	0.361 (0.244,0.489)	0.001	–	–	–
**Intention**	0.386 (0.28,0.482)	0.002	0.386 (0.28,0.482)	0.002	–	–	–

Note. SCD: sudden cardiac death; CPR: cardiopulmonary resuscitation.

## 4 Discussion

Based on the TPB, the extended TPB model was used to investigate the intention of university students to perform bystander CPR on strangers and the factors associated with it. First of all, knowledge, attitude and subjective norm were related to students' intention to perform CPR on strangers, and knowledge could also indirectly affect behavioral intention by influencing subjective norm and attitude. Secondly, our results suggested that students' intention to perform bystander CPR on strangers was mainly determined by their knowledge, attitude, and subjective norm, and perceived behavioral control was not the main determinants. Studies [19] had shown that people with experience performing bystander CPR and those who believed they were capable of performing bystander CPR correctly were more likely to perform bystander CPR. Similarly, we found that “family members of health care workers” and “prior CPR training” were strong determinants of intention to perform bystander CPR, possibly due to increased students’ confidence, but further research was needed. In addition, “gender” and “mastery of CPR related skills” had no significant direct effect on behavioral intention, but might indirectly affect behavioral intention. In conclusion, it is worth noting that this study provides only an exploratory result, and further research and discussion is needed to draw more specific conclusions.

In New England, Magid [25] and his team applied TPB theory to a private university sample and, consistent with their findings, attitude and subjective norm were important predictors of bystander CPR among university students. At the same time, we found that knowledge might also determine students' willingness to act to some extent, which was consistent with the findings of Karuthan's team [26]. It showed that increasing CPR training, improving students' attitude and strengthening social norm could increase students' intention to perform bystander CPR. Studies had shown [27] that a number of behavioral factors might limit students' willingness to perform CPR, including lacking of confidence, fearing of legal action, fearing of disease transmission and embarrassment. Therefore, addressing these concerns and the provision of support to potential bystanders may further encourage bystander CPR.

Despite the various efforts that have been implemented to increase the rate of bystander CPR, many of these initiatives have failed to take into account the TPB. As a result, this study took a theoretical approach to explore the factors that determine the intention of university students to perform bystander CPR. Based on our findings, it is recommended that future CPR training programs for university students focus on improving their attitude towards bystander CPR and the subjective norm surrounding this concept. By doing so, we can increase their intention to perform CPR when the situation calls for it. To further ensure that all students have a good understanding of CPR, we suggest that CPR training should be mandatory for all university students. This will ensure that everyone has the knowledge and skills to potentially save a life in an emergency situation. Finally, it is important to improve positive social norms surrounding bystander CPR. Encouraging students to perform CPR on strangers in need during critical situations can have a significant impact on increasing bystander CPR rates. Therefore, we recommend implementing strategies that promote positive social norms to encourage individuals to take action when needed.

This study has several limitations that should be taken into consideration. First, some factors were neglected in this study, such as the recognition of where the AED is equipped, or to the time until the ambulance arrives. Then, while the sample size of 575 is larger [[28], [29], [30]] than some TPB studies on university students, it is still smaller [31,32] than others. Therefore, future studies should aim to increase the sample size to ensure more representative results. Next, we did not exclude respondents who had already completed the pre-experimental questionnaire, which could have introduced bias into the formal questionnaire survey. Besides, the survey was conducted using an online questionnaire and all respondents participated voluntarily, which might lead to selection bias. The results of the students surveyed may not be representative of the students who did not voluntarily complete the questionnaire. In the future, Offline questionnaires should be used in future research to increase the credibility of the findings. In addition, we included a disproportionate number of female respondents and undergraduate respondents, and given these limitations, our sample of 575 students may not be fully representative of university students in China. Overall, it is important to acknowledge these shortcomings and actively work to address them in future studies.

## 5 Conclusion

This research aims to explore the motives and barriers behind university students' intention to execute bystander CPR, which is a key life-saving skill. By utilizing the TPB as a theoretical framework, this study examined the cognitive and emotional factors that influenced students' attitudes toward conducting CPR as well as subjective norm and perceived behavioral control that affected their behavior. Additionally, the research investigated the impact of demographic factors, such as age, gender, and educational background, on students' CPR competence and intention. The ultimate goal of the study is to provide useful insights for promoting and enhancing CPR training programs among university communities, thereby increasing the probability of successful bystander CPR interventions when needed.

## Funding

This program was supported by the Science and Technology of 10.13039/501100007957Chongqing Education Commission [KJQN202013201].

## Availability of data and materials

Authors can provide all of datasets on reasonable request.

## Consent for publication

Not applicable.

## CRediT authorship contribution statement

**Lihua Xia:** Writing – review & editing, Writing – original draft, Investigation, Funding acquisition, Conceptualization. **Kebiao Zhang:** Writing – review & editing, Supervision, Formal analysis. **Feiyue Huang:** Visualization, Methodology, Formal analysis. **Ping Jian:** Methodology, Formal analysis. **Runli Yang:** Project administration, Investigation, Data curation, Conceptualization.

## Declaration of competing interest

The authors declare the following financial interests/personal relationships which may be considered as potential competing interests: Lihua Xia reports financial support was provided by the Science and Technology of 10.13039/501100007957Chongqing Education Commission. If there are other authors, they declare that they have no known competing financial interests or personal relationships that could have appeared to influence the work reported in this paper.
